# Dot Phrase Utilization and Opioid Prescription at Discharge for Subarachnoid Hemorrhage Patients

**DOI:** 10.1177/19418744261492452

**Published:** 2026-09-28

**Authors:** Natalie Neale, Rebecca DiBiase, Nerissa Ko, Derrick Cheng, Shaadi Settecase, Maggie Waung, Rachel Vassar

**Affiliations:** 1 Department of Neurology, University of California, San Francisco, CA, USA

**Keywords:** subarachnoid hemorrhage, quality improvement, opioid analgesics

## Abstract

**Background and Purpose:**

Subarachnoid hemorrhage (SAH) frequently causes headaches that debilitate patients in the intensive care unit and persist for many patients in follow-up. Current literature suggests that opioids are ineffective for treating SAH pain despite their widespread use and perceived efficacy. There is evidence that implementation of dot phrases in note templates and electronic medical record alerts can positively influence prescriber behavior, including with reducing opioid prescriptions in the primary care setting.

**Methods:**

We assessed rates of opioid prescription in our SAH population and added a standardized dot phrase to admission and discharge summaries. This dot phrase prompted providers to answer whether the patient was being prescribed opioids, the rationale for this, and the plan for weaning.

**Results:**

There was a trend towards overall reduced opioid prescription at discharge after dot phrase implementation. On analysis of the whole cohort, in cases where the dot phrase was used, the rate of opioid prescription at discharge was 14% versus 42% without (chi-square *P* = 0.024). However, dot phrase usage was higher on the neurovascular service compared to neurosurgery (77% vs 39%, Fisher’s exact *P* = 0.043), and the neurovascular service had lower opioid prescription at discharge overall compared to neurosurgery (6% vs 43%, Fisher’s exact *P* = 0.007).

**Conclusions:**

Our results highlight differences in prescription patterns among admitting services for patients with SAH. The results suggest that dot phrase implementation could help reduce opioid prescription at discharge for this patient population, but this may be confounded by higher rates of dot phrase usage on the neurovascular service.

## Introduction

Subarachnoid hemorrhage (SAH) is associated with debilitating headaches in the acute period that in some patients, may persist in long-term follow-up. The pathophysiology of SAH headache pain is complex with several potential implicated factors, including vascular nociception, meningeal irritation, inflammatory response, oxidative damage, superficial siderosis, increased intracranial pressure, mood changes, and N-methyl-D-aspartate (NMDA) receptor upregulation.^1^ One study of 46 patients with SAH found that 89% reported severe pain and that 63% experienced 10/10 pain at some point during their hospitalization.^2^ In a study of 93 patients with SAH, 41% of patients still reported burdensome headache at a mean 33-month follow-up.^3^ Another study found that, even with a median follow-up of 7.5 years, headache frequency was higher in patients with SAH than in controls.^4^ Given this, strategies to improve headache management for SAH patients, both during hospitalization and in follow-up, are warranted. Headache control is also particularly important in the acute period of SAH given pain alleviation can decrease sympathetic tone and help prevent spikes in intracranial pressure.

Current literature suggests that opioids are ineffective for treating SAH pain despite their widespread use, both acutely and chronically, and perceived efficacy by providers.^5-7^ In a comprehensive review, only one study of 22 patients showed an improvement in pain score with opioids, specifically in patients who received fentanyl post-operatively from surgical aneurysm clipping,^8^ while other observational studies of SAH patients receiving daily opioids in the hospital did not demonstrate improvements in pain scores.^1,2,9,10^ Furthermore, opioids in general are associated with severe adverse effects including sedation (limiting neurologic assessments), delirium, rebound headache, bowel dysfunction, and risk of dependence.^11-14^ Opioid prescription at discharge has also been linked to risk of persistent use in the outpatient setting.^15^ SAH patients with certain pain trajectories, such as those with increasing pain in the first week post-hemorrhage, may be at increased risk of long-term opioid use.^16^ Given the lack of strong evidence to support opioid use in treating SAH headache pain and the adverse effects of opioids demonstrated in several populations, it is prudent to consider alternative treatments.

From a hospital perspective, factors that may contribute to opioid over-prescription in SAH patients, including at our hospital, are involvement of multiple care teams, lack of standardized pain medication order-sets among different admitting services, familiarity with prescribing opioids particularly in the intensive care unit setting, and understandable need to aggressively treat pain to prevent hypertension before an aneurysm is secured. There is evidence that dot phrase implementation and electronic medical record alerts can positively influence physician behavior, including with reducing opioid prescriptions in the primary care setting.^17,18^ We aimed to reduce opioid prescription in our primarily aneurysmal SAH population using a dot phrase that prompted providers to reflect on their rationale for prescribing opioids.

## Methods

We collected longitudinal data on our admitted SAH patients sequentially from July 2023 through December 2024 at our tertiary care hospital in San Francisco, California. We included all patients discharged with diagnosis of SAH during this period. We excluded patients who deceased in the hospital. In addition to data on rates of opioid prescription, we collected demographic and clinical data including sex, age, hospital length of stay, modified Fisher score, Hunt and Hess score, admitting service, type of surgical intervention (open clipping versus endovascular treatment for those with aneurysmal source), source of SAH, anterior circulation location (if aneurysmal source identified), size of aneurysm (if aneurysmal source identified), rates of adjunct non-opioid pain medication prescription, rates of pre-existing anxiety or depression, and rates of opioid prescription prior to admission. At our hospital, SAH patients are always admitted to the neurologic intensive care unit initially, and the primary team can be either the neurovascular or neurosurgery service, depending on who is consulted on the case first. Nursing at our institution typically assesses pain on a 0-10 scale for verbal patients, and with the Critical-Care Pain Observation Tool for non-verbal patients, which considers facial expression, movement, muscle tension, and ventilator compliance or vocalization.

In January 2024, we added a standardized dot phrase to admission and discharge summaries for our SAH patients. This dot phrase prompted providers to answer whether the patient was being prescribed opioids, the rationale for this, and plan for weaning (Figure 1). The dot phrase was created by a multidisciplinary team which included members from the neurosurgical and neurovascular services. We did not add this dot phrase to daily progress note templates in order to minimize chart burden. In addition to the dot phrase, we presented our project to unit nursing leadership, informally educated our colleagues on alternative pain control options, and sent email reminders about our initiative.

**Figure 1.**
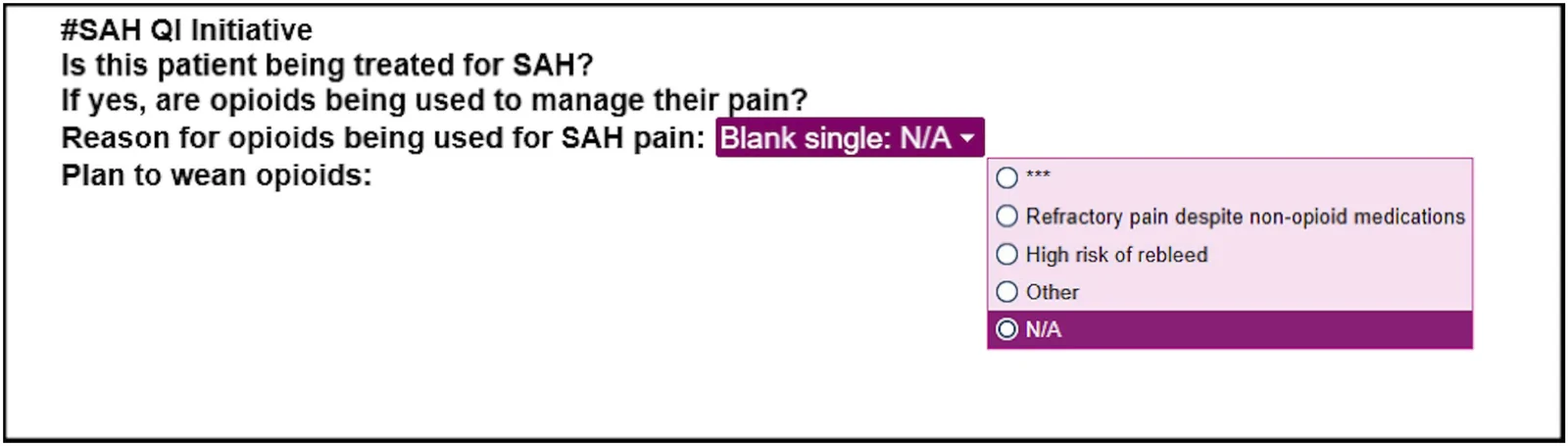
Dot phrase for our quality improvement initiative. SAH = subarachnoid hemorrhage. QI = quality improvement

We also collected data on whether patients were still on opioids at 1-2 months post-discharge, via chart review of outpatient notes. Patients were included in this analysis if there was a primary care, neurology, neurosurgery, or neurointerventional radiology outpatient note within the 30-60 day period post discharge.

This study was determined to be exempt from International Review Board review, as data collection was exclusively based on retrospective chart review with no subject contact and was for quality improvement purposes.

## Data Analysis

For our cohort characteristics, we reported continuous variables with medians and interquartile ranges and categorical variables as percentages. Statistics were performed in Stata Statistical Software: Release 19.5 (College Station, Texas). Categorical variables were compared using Pearson's chi-square tests or Fisher’s exact tests, and Wilcoxon rank-sum tests were used for continuous variables. We performed a logistic regression analysis to assess the relationship between dot phrase and opioid prescription at discharge which included select clinical variables (aneurysmal source, Hunt and Hess score, open intervention), as well as an additional sensitivity analysis that also included admitting service.

## Results

Characteristics of our cohort of SAH patients discharged from our neurovascular or neurosurgery services from July 2023 to December 2024 are shown in Table 1, as well as stratified by pre- and post-dot phrase implementation. The two groups were similar for all baseline characteristics, except a significantly higher median modified Fisher score and Hunt and Hess score in the post-implementation group. Of note, data points are hospitalizations (n = 71) rather than individual patients, as there were two cases of re-admission for re-rupture in our cohort. Median age at hospitalization was 54 years (*IQR* 24), and 26 (37%) of the hospitalizations were male patients. Median length of stay was 16 days (*IQR* 10), median Hunt and Hess score was 2 (*IQR* 2), and median modified Fisher score was 3 (*IQR* 3). In 9 (13%) of the encounters, open surgical clipping of the aneurysm took place. In 51 (72%) of the encounters, an aneurysmal source was identified. Of the 20 cases where no aneurysmal source was identified, 7 were perimesencephalic hemorrhage, 3 were likely traumatic in retrospect, 1 was thought to be secondary to cerebral amyloid angiopathy, 1 was thought to be reversible cerebral vasoconstriction syndrome, 1 was secondary to arteriovenous malformation, and the remainder were of unclear etiology. In our cohort, in 5 (7%) of the encounters, patients were prescribed opioids prior to admission, and 10 (14%) had pre-existing anxiety or depression. In our cohort overall, in 24 (34%) encounters patients were discharged with opioids, and in 69 (97%) encounters patients were discharged with non-opioid pain medication.

**Table 1.** Cohort Characteristics, Overall and Pre- and Post-Dot Phrase Implementation

	Total cohort	Pre-dot phrase implementation	Post-dot phrase implementation	*P*-value
Total encounters	71	30	41	
Median age in years (IQR)	54 (24)	58 (25)	51 (25)	0.545
Total male	26 (37%)	12 (40%)	14 (34%)	0.613
Total on neurovascular service	17 (24%)	4 (13%)	13 (32%)	0.095
Median length of stay in days (IQR)	16 (10)	14 (9)	19 (11)	0.069
Median Hunt and Hess score (IQR)	2 (2)	1.5 (1)	2 (1)	<0.001
Median modified Fisher score (IQR)	3 (3)	2.5 (2)	3 (2)	0.037
Total with identified aneurysmal source	51 (72%)	21 (70%)	30 (73%)	0.769
Anterior circulation aneurysm if aneurysm identified	39 (76%)	19 (90%)	20 (67%)	0.091
Median size of aneurysm in millimeters if aneurysm identified (IQR)	5 (5)	6.6 (4.1)	4.3 (4.5)	0.144
Total open intervention	9 (13%)	3 (10%)	6 (15%)	0.724
Total discharged with opioids	24 (34%)	13 (43%)	11 (27%)	0.146
Total discharged with non-opioid adjunct therapy	69 (97%)	30 (100%)	39 (95%)	0.505
Total prescribed opioids prior to admission	5 (7%)	2 (7%)	3 (7%)	1.000
Total exposed to opioids in hospital	71 (100%)	30 (100%)	41 (100%)	1.000
Total exposed to intravenous opioids in hospital	69 (97%)	29 (97%)	40 (98%)	1.000
Total prescribed non-opioid pain medication in hospital	69 (97%)	29 (97%)	40 (98%)	1.000
Total with pre-existing anxiety or depression	10 (14%)	4 (13%)	6 (15%)	1.000

Abbreviations: IQR = interquartile range.

In regard to admitting service, 17 (24%) were admissions to the neurovascular service and the remainder were admissions to the neurosurgery service. Baseline characteristics did not vary significantly between admitting services, including rates of open intervention (Supplemental Table 1). Rate of opioid exposure during hospitalization was similar between services (100% on neurovascular, 100% on neurosurgery, Fisher’s exact *P* = 1.000). Opioid prescription at discharge, however, was significantly higher among patients on the neurosurgical service (43% compared to 6%, Fisher’s exact *P* = 0.007). We found that dot phrase usage varied significantly by service (10/13 or 77% for neurovascular service, and 11/28 or 39% for neurosurgical service, Fisher’s exact *P* = 0.043). Rates of opioid prescription on the neurosurgical service were 50% (13/26) pre-dot phrase implementation, and 36% (10/28) post-implementation (Fisher’s exact *P* = 0.409). For the neurovascular service, rates of opioid prescription were 0% (0/4) in the pre-implementation subgroup and 8% (1/13) in the post-implementation subgroup (Fisher’s exact *P* = 1.000). Within the post-implementation subgroup, for the neurovascular service, 0% (0/3) were discharged with opioids if dot phrase was not included, and 10% (1/10) were discharged with opioids with dot phrase included (Fisher’s exact *P* = 1.000). Among the neurosurgical patients in the post-implementation subgroup, 47% (8/17) were discharged with opioids if dot phrase was not included, and 18% (2/11) were discharged with opioids if dot phrase was included (Fisher's exact *P* = 0.226).

Within our whole cohort, we analyzed rates of opioid prescriptions pre and post-dot phrase implementation in January 2024, regardless of dot phrase use. Rate of opioid prescription at discharge pre-dot phrase was 43% (13/30), compared to 27% (11/41) post-dot phrase implementation, though the difference was not statistically significant (chi-square *P* = 0.146, Figure 2). Among those with dot phrase, opioid prescription was 14% at discharge, compared to 42% for those without dot phrase for the whole cohort (chi-square *P* = 0.024). Within the post-implementation subgroup, opioid prescription was lower in the dot phrase group but not statistically significant (Table 2). Those with dot phrase included were more likely to be on the neurovascular service and to have an aneurysmal source identified.

**Figure 2.**
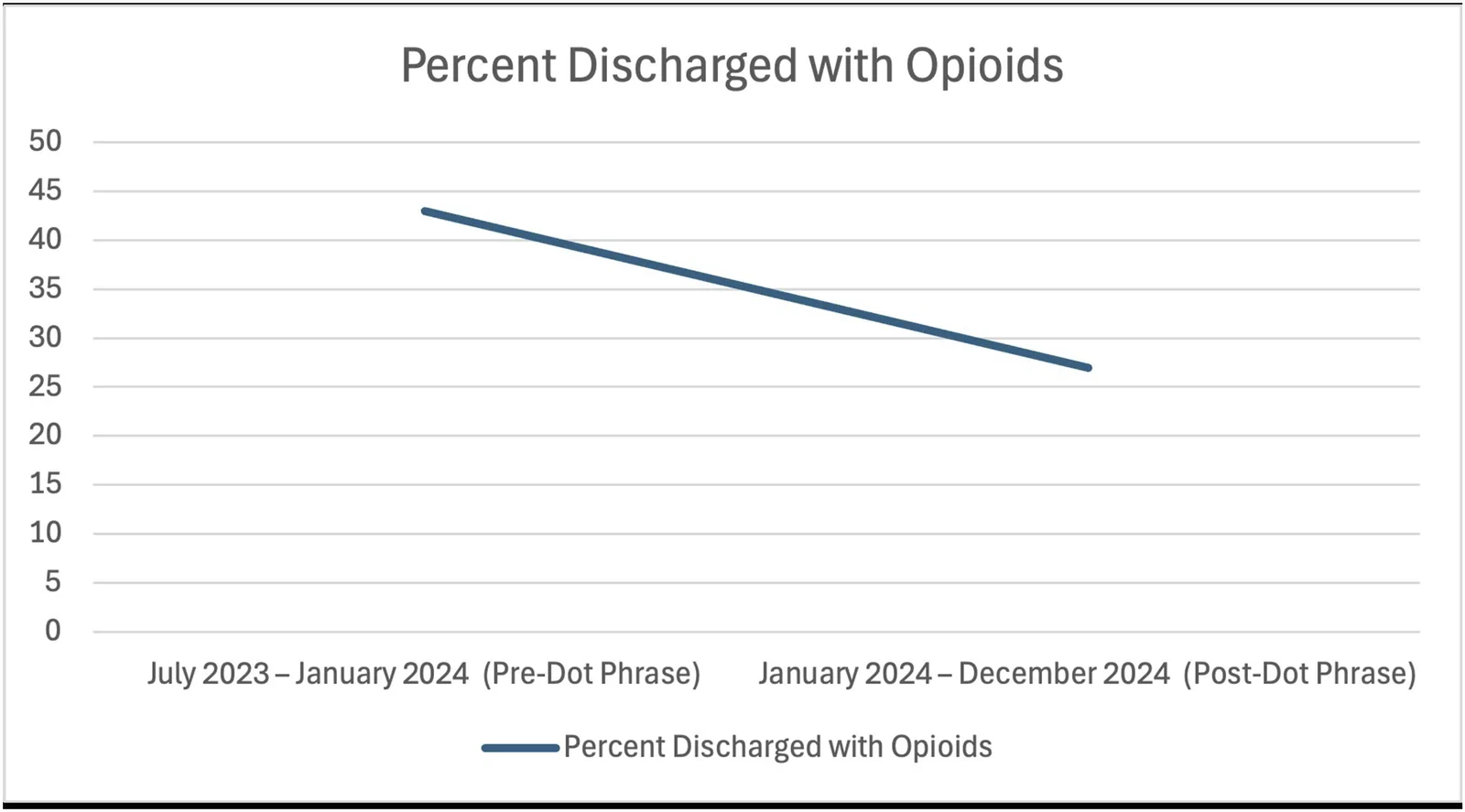
Rates of opioid prescription at discharge.

**Table 2.** Post-Dot Phrase Rates of Opioid Prescription and Clinical Characteristics, Stratified by Dot Phrase Use

​	Dot phrase included	Dot phrase not included	*P*-value
Total encounters	21	20	​
Median age in years (IQR)	50 (33)	55 (19)	0.240
Total male	6 (29%)	8 (40%)	0.440
Total on neurovascular service	10 (48%)	3 (15%)	0.043
Median length of stay in days (IQR)	19 (16)	19 (6)	0.865
Median Hunt and Hess score (IQR)	2 (1)	2.5 (2)	0.168
Median modified Fisher score (IQR)	3 (3)	3.5 (1)	0.160
Total with identified aneurysmal source	19 (90%)	11 (55%)	0.015
Anterior circulation aneurysm if aneurysm identified	14 (74%)	6 (55%)	0.425
Median size of aneurysm in millimeters if aneurysm identified (IQR)	5 (4.5)	4 (6.5)	0.618
Total open intervention	3 (14%)	3 (15%)	1.000
Total discharged with opioids	3 (14%)	8 (40%)	0.063
Total discharged with non-opioid adjunct therapy	19 (90%)	20 (100%)	0.488
Total prescribed opioids prior to admission	2 (10%)	1 (5%)	1.000
Total exposed to opioids in hospital	21 (100%)	20 (100%)	1.000
Total exposed to intravenous opioids in hospital	21 (100%)	19 (95%)	0.488
Total prescribed non-opioid pain medication in hospital	20 (95%)	20 (100%)	1.000
Total with pre-existing anxiety or depression	3 (14%)	3 (15%)	1.000

On logistic regression analysis of the cohort as a whole including select clinical variables (aneurysmal source, type of intervention, and Hunt and Hess score), dot phrase inclusion remained associated with lower rates of opioid prescription at discharge (adjusted OR 0.20; 95% CI 0.05-0.82; *P* = 0.026). On additional sensitivity analysis, we added admitting service to this model, and association of dot phrase with lower odds of opioid prescription at discharge did not retain significance (adjusted OR 0.34; 95% CI 0.07-1.55; *P* = 0.163).

Opioid prescription prior to admission was not associated with new opioid prescription at discharge (20% or 1/5 among those with opioid prescription prior to admission vs 35% or 23/66 among those without, Fisher’s exact *P* = 0.656). Pre-existing anxiety or depression was also not associated with new opioid prescription at discharge (30% or 3/10 among those with anxiety or depression vs 34% or 21/61 among those without, Fisher’s exact *P* = 0.546).

On our follow-up analysis, 43 patients had clinic notes identified in the chart from 30-60 days post-discharge from either primary care, neurosurgery, neurology, or neurointerventional radiology. Of these, 7 were on opioids based on chart review: 2 were on the same opioid they were prescribed prior to admission, and the remaining 5 had been prescribed a new opioid at discharge.

## Discussion

After implementing a dot phrase at our tertiary care center prompting providers to reflect on opioid prescription in SAH patients, we noted a trend of decreased opioid prescription at discharge from 43% to 27%, though not statistically significant. Rates of opioid prescription at discharge were significantly lower in patients where dot phrase was included (14% compared to 42% without) within the whole cohort, though not within the post-implementation subgroup. Dot phrase usage was higher among patients admitted to the neurovascular service, which also had a lower rate of opioid prescription at discharge overall. Our cohort had a female predominance and a median age of 54 years, consistent with the demographics of non-traumatic SAH reported in other studies.

Given the over-prescription of opioids in this population, other methods of pain control should be considered. Alternative treatments for SAH-related headache that have promising evidence in small studies include gabapentinoids, occipital nerve blocks, pterygopalatine fossa blockade, and intravenous lidocaine infusion.^19-23^ In a case series, sumatriptan was found to reduce pain scores when inadvertently given to patients with SAH, but its use in this population is limited by theoretical risk of vasospasm.^24^ Nonsteroidal anti-inflammatory drugs (NSAIDs) have historically not been used out of concern for increasing bleeding in this population, but recent data suggests NSAIDs may be safe in patients post-operatively after craniotomy, particularly in the subacute period.^25^ While evidence for acetaminophen in aneurysmal SAH is limited, its safety profile is favorable to opioids and it is commonly used.^2^ Treatment options that have not yet been studied but may have promise include calcitonin gene-related peptide inhibitors, tricyclic antidepressants, cannabinoids, and minocycline.^1^ At our institution, we commonly use acetaminophen and gabapentinoids as adjunct pain medications. While treating SAH-related headache remains challenging, evidence for some non-opioid treatment strategies should prompt physicians to consider multimodal opioid-sparing pain control. While our cohort had high rates of adjunct non-opioid pain medication prescriptions, the fact that there was still a proportion discharged with opioids highlights the need for better optimization of these adjunct therapies.

Our initiative suggests that dot phrase implementation into note templates may be a high-yield, low-effort, and low-cost way to modify physician behavior in regard to opioid over-prescription, which remains an issue in the SAH population. Our study also highlights a potential need for targeted education on SAH multimodal pain management for provider groups that have different inpatient care practices. While our primary focus was on opioid prescription at discharge, our post-discharge follow-up analysis also suggests that new opioids were not prescribed in the short-term for those discharged without opioids. We are not aware of any other studies focusing on quality improvement initiatives to reduce opioid prescriptions in subarachnoid hemorrhage. However, education initiatives, standardized protocols, and electronic health record alerts have decreased opioid prescription in other post-operative populations.^26,27^ For example, in an otolaryngologic surgery population, implementation of standardized discharge instructions in the electronic medical record helped reduce opioid prescription.^28^ In our study, we suspect that the dot phrase was responsible for the decrease in opioid prescriptions more so than our informal education efforts, but both may have been impactful, and more formal education initiatives in this area could potentially further decrease opioid prescriptions. The rise of artificial intelligence and machine learning may also allow for better prediction and identification of patients at highest risk for developing long-term opioid use.^29^ There will likely be more opportunities to leverage technology for quality improvement as these advancements continue to integrate into our medical practice.

## Limitations

One challenge of this initiative was the lack of universal adoption of the dot phrase, likely related to chart burden and variability in templates used by providers. While we purposefully made our dot phrase short to minimize effort required by the provider, we acknowledge that time spent in the medical record is associated with physician burnout,^30^ and even a small amount of extra charting can contribute to this. This is perhaps particularly relevant to busy surgical services, where there may be temptation to delete the dot phrase due to time constraint, and in our study the neurosurgery service had a lower rate of dot phrase uptake. Wider note template variation used by the neurosurgical service providers may have also contributed. Even within the neurovascular service, we were not able to make the dot phrase appear for only SAH patients, which likely contributed to some charting burden and dot phrase completion fatigue. An electronic medical record function that is incorporated into the discharge workflow with a hard stop may be a more impactful approach, but the process of creating this is much more labor-intensive than creating a dot phrase.

We also acknowledge that there may be differences in opioid prescribing likelihood of providers who choose to use the dot phrase, which may confound the results. For example, in our study, the neurovascular service had high rates of dot phrase usage, and this service had lower rates of opioid prescription at discharge in the overall cohort. Thus, the lack of service uniformity in our study may be a limitation. However, because our study included both neurovascular and neurosurgery service patients, we were able to sample across a more heterogenous group of providers and therefore results may be more broadly applicable. We also acknowledge that the difference in rates of opioid prescription pre- and post-dot phrase implementation could be related to our other efforts such as informal education and increased awareness towards the issue. However, our project did not include robust, formal educational efforts, which may potentially have had a greater impact on prescriber behavior.

Other limitations of our study include our relatively small sample size, and our lack of longer-term follow-up. Our results should be interpreted cautiously because while dot phrase usage was associated with lower opioid prescriptions compared to those without dot phrase in the cohort overall, this association was not seen when looking only at the post-implementation subgroup, perhaps due to being underpowered. We included a short-term follow-up analysis, but longer interval follow-up may have been more informative regarding opioid dependence. Our follow-up data was also collected via chart review rather than contacting patients directly, and many patients in the cohort did not have follow-up data for the timeframe analyzed. Inferring opioid use from notes and medication list reviews may also give a less complete picture than direct patient interview.

Our project also primarily focused on opioid prescription at discharge, but as discussed previously, opioid over-prescription in the hospital is also a significant problem that should be addressed. In our cohort, the vast majority received intravenous opioid medication in the hospital, though in many cases this could have been for indications unrelated to headache (such as periprocedural pain or ventilator synchrony). More concerted efforts to target opioid reduction earlier in admission would likely also reduce opioid prescription at discharge and have a positive impact on patient care. Finally, our population in this study was also primarily aneurysmal SAH, so results are likely not generalizable to primarily traumatic SAH populations. Nonetheless, our results provide evidence that quality improvement initiatives such as dot phrases may decrease opioid prescription at discharge in certain SAH patients, though results are limited by dot phrase uptake and differences in prescribing patterns among services.

## Supplemental Material

Supplemental Material Dot Phrase Utilization and Opioid Prescription at Discharge for Subarachnoid Hemorrhage Patients Supplemental Material for Dot Phrase Utilization and Opioid Prescription at Discharge for Subarachnoid Hemorrhage Patients by Natalie Neale, Rebecca DiBiase, Nerissa Ko, Derrick Cheng, Shaadi Settecase, Maggie Waung, Rachel Vassar, in The Neurohospitalist

## Data Availability

Data are available from the corresponding author upon reasonable request.*
