# Supplementary material for: Dot Phrase Utilization and Opioid Prescription at Discharge for Subarachnoid Hemorrhage Patients
Source: Neurohospitalist. 2026 Sep 28:19418744261492452. Online ahead of print. doi: 10.1177/19418744261492452 (PMC13619976; doi:10.1177/19418744261492452)
Supplement: Supplementary file 1 [file sj-pdf-1-nho-10.1177_19418744261492452.pdf]

Supplemental Table 1: Baseline characteristics stratified by admitting service.

Variable	Neurovascular Service	Neurosurgery Service	P-value
**Median Age in Years (IQR)**	51 (24)	55 (24)	0.787
**Total Male**	7 (41%)	19 (35%)	0.655
**Median Length of Stay in Days (IQR)**	18 (9)	15 (13)	0.928
**Median Hunt Hess Score (IQR)**	2 (2)	2 (2)	0.623
**Median Modified Fisher Score (IQR)**	3 (2)	3 (3)	0.454
**Total With Identified Aneurysmal Source**	13 (76%)	38 (70%)	0.762
**Anterior Circulation Aneurysm if Aneurysm Identified**	12 (92%)	27 (71%)	0.151
**Median Size of Aneurysm in Millimeters if**	6 (3)	4.5 (5.15)	0.131

**Aneurysm Identified (IQR)**			
**Total Open Intervention**	3 (18%)	6 (11%)	0.439
**Total Prescribed Opioids Prior to Admission**	2 (12%)	3 (6%)	0.587
**Total With Pre-Existing Anxiety or Depression**	3 (18%)	7 (13%)	0.694
